# Clinical value of nano‐carbon lymphatic tracer for regional lymph node dissections of rectal cancer after neoadjuvant chemoradiotherapy

**DOI:** 10.1002/acm2.14406

**Published:** 2024-05-31

**Authors:** Feng Shao, Qi Zhou, Fei Yu, Lelin Pan, Lijun Li

**Affiliations:** ^1^ Department of Anorectal surgery Dongyang People Hospital (affiliated Dongyang Hospital of Wenzhou Medical University) Dongyang, Zhejiang province China; ^2^ The First Affilated Hospital Zhejiang University School of Medicine Qingchunlu Zhejiang China

**Keywords:** lymph node, nano‐carbon, pathological staging, rectal cancer, tracer

## Abstract

**Objectives:**

Regional lymph node (LN) volume decreases after neoadjuvant therapy, requiring a tracer for more accurate detection. Nano‐carbon tracer is a third‐generation tracer with several advantages, but its use for LN detection after neoadjuvant chemoradiotherapy for middle and low rectal cancer remains unclear. Therefore, this study investigated the effects and safety of anoscope‐guided subrectal injections of nano‐carbon suspension in this patient population.

**Methods:**

This study retrospectively reviewed the medical records of 45 patients with middle and low rectal cancer admitted to our institution from March 2019 to March 2022. All patients received preoperative neoadjuvant chemotherapy and radiotherapy and were divided into nano‐carbon injection (*n* = 23; anoscope‐guided injections of nano‐carbon suspension in the rectal submucosa 2 cm above the dentate line 24 h preoperatively) and control (*n* = 22; directly underwent surgery) groups. The LN detection and complication rates were compared between the groups.

**Results:**

The total and mean numbers of LNs and small LNs and the number of patients with > 12 LNs were significantly higher in the nano‐carbon injection group than in the control group. The total number of positive LNs and LN metastasis did not differ between the groups, nor did the anastomotic leakage, bleeding, stenosis, and abscess occurrence rates.

**Conclusions:**

Anoscope‐guided nano‐carbon lymphatic tracing increased the LN detection rate, caused less trauma, and resulted in fewer postoperative complications than the direct surgical procedure. Thus, it is an effective, safe, and practical method that may improve dissections and the postoperative pathological staging accuracy.

## 1 INTRODUCTION

Colorectal cancer is a malignant tumor affecting the digestive system with increasing global morbidity and mortality rates,^1^ and neoadjuvant chemoradiotherapy and radical surgery are the most common treatments for locally advanced middle and low rectal cancer with good curative effects.^2^ The number of resected lymph nodes (LNs) is an effective measure of the radical resection of rectal cancer; for example, insufficient LNs during the postoperative pathological examination can lead to inaccurate staging, increasing the risk of postoperative recurrence.^3^ Neoadjuvant therapy shrinks regional LN volume, making LN detection more challenging,^4^ often requiring colonoscope‐guided injections of LN tracers around the tumor to improve the detection rate,^5^ but this also affects tissue dissection during the operation. Nano‐carbon suspension is a third‐generation LN tracer (a biological dye) with high specificity, good safety, and good lymphatic tendency^6^ that does not cause anaphylaxis in humans.^7^ This type of lymph node tracer is widely used in thyroid or breast surgery. When nanocarbon is injected into the surrounding tissue of the tumor during surgery, it will spread along the lymphatic ducts and lymph nodes, and the lymph nodes will turn into black nodules to distinguish them from normal tissue. At the same time, posterior visualization of the parathyroid gland can be performed, making it easier to protect the parathyroid gland during surgery to avoid postoperative calcium deficiency. Nanocarbon is a good lymph node tracer, which can better display lymph nodes and perform more thorough lymph node dissection during surgery, but its use for LN detection in patients with low and middle rectal cancer remains unclear. Therefore, this study explored the value and safety of anoscope‐guided nano‐carbon injections in patients undergoing laparoscopic anterior rectal cancer resection after neoadjuvant therapy.

## 2 MATERIALS AND METHODS

### 2.1 Data collection and patients

We retrospectively analyzed the clinical data of patients with middle and low rectal cancer admitted to our institution from March 2019 to March 2022. Patients (1) with a pathological diagnosis of rectal adenocarcinoma; (2) a rectal mass 4–12 cm away from the anus (i.e., middle and low rectal cancer; (3) tumor (T) stage 3 or 4 or LN metastasis based on pelvic magnetic resonance imaging, thus, meeting the criteria for neoadjuvant therapy; (4) without lung, liver, or other distant metastasis; and (5) without surgical contraindications (e.g., major underlying diseases or coagulation disorders) were included. The patients were randomly numbered and divided into an odd and even number into a nano‐carbon injection group and a control group to ensure the randomness of the grouping. All patients provided informed consent for the nano‐carbon injections before surgery. This study has been approved by our review committee (IRB approval number: KY 2018‐003‐01). All subjects or their legal representatives sign a written informed consent form to collect clinical information upon admission.

### 2.2 Research design

All patients underwent preoperative neoadjuvant therapy; Using Linear Accelerator (Elekta Axesse, Elekta Solutions AB, Sewden) for X‐ray radiation therapy, the total radiotherapy dose was 45–50 Gy, with 1.8−2.0 Gy doses per session five times per week for 5 weeks. Synchronous chemotherapy was orally administered twice daily (capecitabine, 1250 mg/m^2^). The feasibility of radical rectal cancer resection was evaluated 5−12 weeks after concluding the neoadjuvant therapy.

**FIGURE 1:**
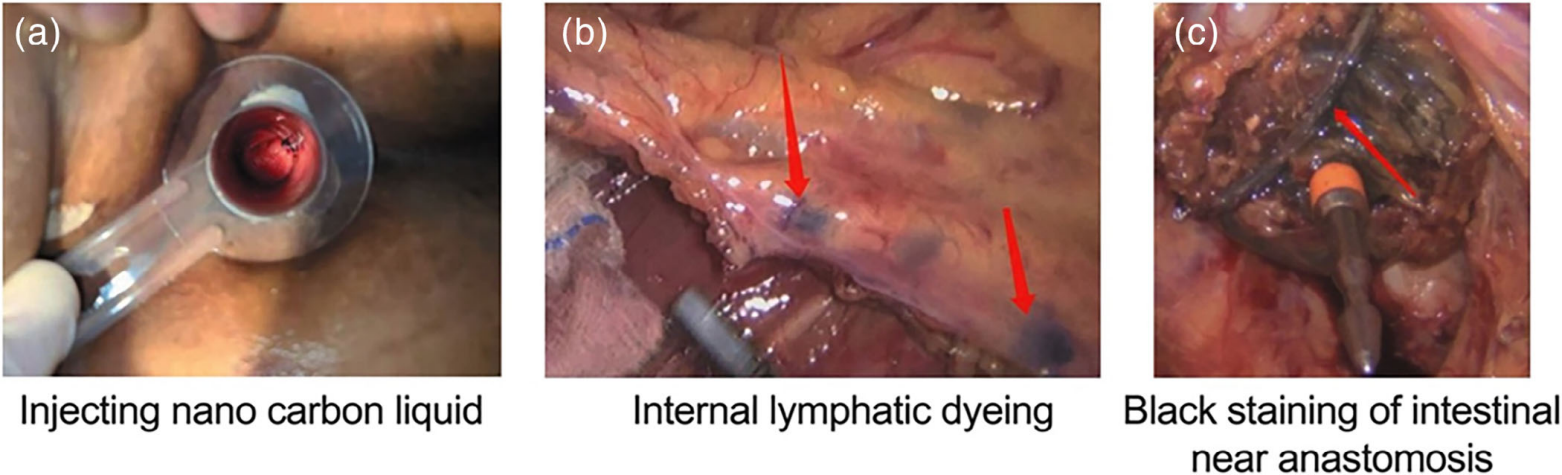
Representative images of nano‐carbon tracer injections for lymph node resections. (a) Nano‐carbon liquid injection. (b) Internal dyeing of the lymph nodes (red arrows). (c) Black staining of the tumor site (near intestinal anastomosis; red arrow) after nano‐carbon tracer injection.

Patients in the nano‐carbon injection group received 0.15−0.25 mL injections of nano‐carbon suspension, which is a third‐generation LN tracer with high specificity and good lymphatic tendency, 2 cm from the dentate line 24 h before surgery; they were administered slowly and guided by an anoscope. Injections were administered at three points on the same plane of the intestine. During the injection, care was taken to prevent intestinal wall penetration and contamination of the surrounding tissue (Figure 1a). The nano‐carbon suspension (trade name: Kanalin) was purchased from Chongqing Meilai Pharmaceutical Co., Ltd. (Chongqing, China). The control group did not receive nano‐carbon suspension injections.

All patients underwent laparoscopic anterior rectal cancer resection by the same group of physicians using total mesorectal excision techniques,^8^, ^9^ followed by intestinal resection and anastomosis. Because the pelvic floor region had received radiotherapy, the proximal rectum was transected above the sacroiliac joint level during the operation, and the length of the resected bowel was more than 10 cm to ensure the number of lymph nodes dissected. In addition, all patients had a protective ileostomy. The tumor tissue underwent postoperative pathological evaluations.

### 2.3 Outcomes

The pathological outcomes included the total number of LNs and the numbers of small and positive LNs, as well as the metastasis rate. Additionally, anastomotic complications were assessed 60 days postoperatively, including infection and anastomotic bleeding, leakage, and stenosis.

### 2.4 Statistical analyses

Statistical analyses were performed using SPSS version 24.0 (IBM Corp., Armonk, NY, USA). Enumeration data are represented by *n* (%), and the differences between groups were analyzed by chi‐square or Fisher's exact tests. The Kolmogorov–Smirnov test was used to analyze data distributions. T‐tests were used to compare groups of normally distributed data, expressed as means ± standard deviations. Non‐parametric tests were used to compare non‐normally distributed data, expressed as medians and upper and lower quartiles. *p*‐values of < 0.05 indicated statistically significant differences.

## 3 RESULTS

### 3.1 Patient demographics

This study included 45 patients with rectal cancer; 23 and 22 patients were included in the nano‐carbon injection and control groups, respectively. Preoperative nano‐carbon tracer injections resulted in clearly stained tumor sites (Figure 1). Sex, age, body mass index, the Tumor, Node, Metastasis stage, and other baseline characteristics did not differ between the two groups, indicating they were comparable (Table 1).

**TABLE 1:** Baseline and clinicopathological characteristics.

Index	Tracer group^*^ (*n* = 23)	Control group^*^ (*n* = 22)	χ^2^	*p*‐value
**Sex**			0.67	0.82
Male	13	11		
Female	10	11		
**Age (years)**			0.48	0.93
<60	11	10		
≥60	12	12		
**Body mass index (kg/m^2^)**			0.54	0.82
<24	12	10		
≥24	11	12		
**Tumor stage**			0.93	0.45
Tis	2	3		
T_1_	5	6		
T_2_	6	5		
T_3_	5	4		
T_4_	4	4		

* Data are presented as the number of patients.

### 3.2 LN detection

The total numbers of LNs detected in the nano‐carbon injection and control groups were 359 (average: 15.6 ± 3.1) and 196 (average: 8.9 ± 2.1), respectively. Significantly more LNs were detected in the nano‐carbon injection group than in the control group (*p* < 0.05). In the nano‐carbon injection group, >12 LNs were detected in 22/23 patients (95.7%), which was significantly higher than that in the control group (13/22 patients, 59.1%) (*p* < 0.05). Furthermore, significantly the total numbers of more small LNs were detected in the nano‐carbon injection group than in the control group (total: 117, average: 5.09 ± 2.16 vs. total: 47, average: 2.14 ± 1.16) (*p* < 0.05). Finally, the positive LN detection rate (nano‐carbon: 117 LNs, 21.2% vs. control: 39 LNs, 19.9%) and metastasis rate (nano‐carbon: 18 cases, transfer rate: 78.3% vs. control: 17 cases, transfer rate: 77.3%) did not differ between the two groups. Table 2 provides detailed LN data.

**TABLE 2:** Lymph node detection.

Index	Tracer group (*n* = 23)	Control group (*n* = 22)	t/χ^2^/Z	*p*‐value
**All lymph nodes**				
Total (*n*)	359	196	–	–
Mean ± standard deviation	15.6 ± 3.1	8.9 ± 2.1	9.382	<0.001
Patients with > 12 lymph nodes (*n*)	22	13	14.573	<0.001
**Small lymph nodes (<5 mm)**				
Total	117	47	–	–
Mean ± standard deviation	5.09 ± 2.16	2.14 ± 1.16		<0.001
Minimum (*n*)	2	0		
Maximum (*n*)	11	6		
Positive lymph nodes (*n*)	76	39	–	–
Lymph node metastasis rate	21.2%	19.9%		
Patient transferred (*n*)	18	17	0.0328	0.953

### 3.3 Complications

Table 3 presents postoperative complication data. Neither the tracer group nor the control group showed any signs of infection. There was no significant difference in anastomotic bleeding (1/23vs2/22, *p* = 0.524), anastomotic fistula (2/23vs3/22, *p* = 0.598), and anastomotic stenosis (1/23vs1/22, *p* = 0.974). The complication incidence rate did not differ between the two groups.

**TABLE 3:** Anastomosis‐related complications.

Complication	Tracer group^*^ (*n* = 23)	Control group^*^ (*n* = 22)	χ^2^	*p*‐value
Infection	0	0	0.000	1.000
Anastomotic bleeding	1	2	0.407	0.524
Anastomotic fistula	2	3	0.278	0.598
Anastomotic stenosis	1	1	0.001	0.974

* Data are presented as the number of patients.

### 3.4 Factors influencing positive LN detection in radical rectal cancer resection

A multivariate logistic regression model was used to identify factors affecting the positive detection of LNs (Table 4). The T stage, tissue pathological grade, vascular and nerve invasion, and nano‐carbon tracer were independent influencing factors for positive LN detection in radical rectal cancer surgery (*p* < 0.05).

**TABLE 4:** Multivariate analysis for factors affecting the number of positive lymph nodes after minimally invasive radical resection.

Index	B‐value	SE	χ^2^	OR	95% CI	*p*‐value
Tumor stage 2	22.83	0.54	120.77	5.34	1.48−12.31	<0.001
Tumor stage 3	23.15	0.44	141.32	6.04	1.02−17.83	<0.001
Tumor stage 4	22.91	0.61	131.66	4.12	1.27−8.03	<0.001
Organization level 3−4	1.34	0.37	55.78	4.83	1.52−8.83	<0.001
Vascular invasion	0.72	0.38	23.17	2.93	1.71−11.09	<0.001
Nano‐carbon tracer	1.94	0.43	60.92	7.02	1.57−17.32	<0.001

Abbreviations: CI, confidence interval; OR, odds ratio; SE, standard error.

## 4 DISCUSSION

Neoadjuvant therapy is an effective treatment for locally advanced middle and low rectal cancer, reducing the tumor volume and increasing the anus preservation rate, resulting in negative peripheral resection margins and, thus, improved success rates, survival times, and quality of life.^10^, ^11^ However, radiotherapy‐ and chemotherapy‐induced LN atrophy from lymphocyte depletion, interstitial hyperplasia, and fibrosis^12^ makes LN detection during radical rectal cancer surgery after neoadjuvant therapy difficult, considerably reducing the number of detected LNs. The main substances used for gastrointestinal staining are suspension of carbon particles, including Indian ink, methylene blue, indigo carmine, indocyanine green, toluidine blue, isothiocyan and hematoxylin eosin, nanocarbon, etc. Recent studies have also confirmed that using the patient's own venous blood as a staining agent is also a feasible, simple, and safe method.

At present, nano carbon is widely used in preoperative localization of clinical surgery, such as thyroid, breast cancer, lung cancer, gastric cancer, etc. It is also popular in colorectal cancer surgery. Before surgery, nano carbon suspension can be injected around the tumor through colonoscopy to effectively locate the tumor. Accurate preoperative localization is extremely important for laparoscopic colorectal cancer surgery, especially for the colon. If the tumor cannot be accurately located, the wrong segment may be removed during surgery, because the colon cannot be palpated during laparoscopic surgery. Pre operative localization of nanocarbon is helpful for accurate localization of early colon cancer lesions, thereby improving the safety and accuracy of surgery, especially for laparoscopic surgery. Preoperative injection of nanocarbon can also effectively locate advanced tumors, especially in patients with intra‐abdominal adhesions. Nanocarbon can enable surgeons to quickly locate the lesion, reduce the time spent searching for tumors during surgery, thereby shortening the surgical time and reducing intraoperative bleeding. Numerous studies have found that the accuracy of preoperative application of nanocarbon localization is >90%.

Nano‐carbon LN tracer injections are an effective way to increase the number of detected LNs.^13^, ^14^, ^15^ The diameter of a nano‐carbon particle is consistently 150 µm, which is smaller than the capillary lymphatic endothelial cell gap (120−500 µm). Additionally, the basement membrane is not fully developed and has high permeability, allowing nano‐carbon particles to enter the lymphatic vessel. Furthermore, macrophages phagocytose nano‐carbon, increasing the tropism of the nano‐carbon tracer to lymph, resulting in specific, black staining of the LNs. Ideally, the nano‐carbon suspension is injected 24 h before the operation^16^; if the interval is too short, the regional lymphatic drainage cannot be displayed.^17^, ^18^

The 8th edition of the American Joint Committee on Cancer Staging treatment guidelines recommend at least 12 LNs in the resection specimen after radical rectal cancer surgery to accurately assess tumor staging, make follow‐up treatment and prognostic decisions, and reduce the local tumor recurrence risk, thereby increasing the long‐term survival rate, Fewer than 12 LNs, lower rectal cancer, and stage T3 or T4 are high‐risk factors for lateral LN metastasis.^19^, ^20^, ^21^, ^22^, ^23^ However, only 20% of surgical specimens have 12 or more LNs.^24^ Therefore, increasing the number of LNs detected in the cancer specimens after neoadjuvant therapy is a major challenge for colorectal surgeons. We found that more patients in the nano‐carbon group had > 12 LNs in the resected specimen than in the control group, implying that nano‐carbon injections improve the LN detection rate.

Pathologists often rely on hand touch to detect LNs, which is not very sensitive. Many small LNs are hidden in fat or mesentery and are difficult to identify, resulting in a low detection rate. However, nano‐carbon LN tracers can help pathologists detect LNs in adipose tissue, especially for those < 5 mm in diameter, increasing the detection rate and improving the accuracy of pathological N staging. Studies have demonstrated that long‐term survival increases in patients with stage II and III colon cancer as the number of removed LNs increases.^25^ As more LNs are removed, more metastatic LNs are detected, affecting the pathological N staging, which may explain this result. Moreover, those results emphasize that accurate pathological staging of rectal cancer is helpful for adjuvant treatment and prognostic decisions, improving survival rates. We also found that nano‐carbon injections increased the number of LNs detected in the resected rectal cancer specimen after neoadjuvant treatment. Furthermore, our multi‐variable analysis identified nano‐carbon injections as an independent influencing factor for the number of detected LNs. However, nano‐carbon injections did not affect the number of positive LNs detected.

In our study, the number of small LNs detected in the nano‐carbon group was more than two times higher than that of the control group, indicating that nano‐carbon injections improved the small LN detection rate. Improving small LN detection is important because small lymph nodes are potentially metastatic; thus, assessing them can improve the pathological staging accuracy. Several studies have reported that tumor cell metastasis exists in small LNs, accounting for 45.4% of the total number of positive LNs.^26^, ^27^, ^28^ Moreover, the incomplete dissection of small lesions and metastatic LNs is the primary reason for postoperative recurrence and death in patients with rectal cancer.^29^ Small LN detection improves the pathological N staging accuracy of colorectal cancer, which affects postoperative adjuvant therapy selection. However, small LNs (diameter < 5 mm) are not easily detected. Therefore, improving the detection method, and thus the detection rate, for small LNs is crucial, with a goal of more than 12 LNs in surgical specimens after neoadjuvant therapy.

In this study, anoscope‐guided nano‐carbon injections around the tumor before radical resection of rectal cancer increased the chance of detecting 12 or more LNs in the resected specimen. LN drainage of the recto‐anal canal is bound by the dentate line and divided into upper and lower groups. The upper group, above the dentate line, has three drainage directions: upward (through the superior rectal artery to the side of the inferior mesenteric artery LNs) and to the sides and down (to the LNs next to the internal iliac vessels). Since 2017, our research group has been working to improve the nano‐carbon suspension injection method. Based on the characteristics of lymphatic drainage of middle and low rectal cancer, we chose anoscope‐guided injections into the submucosa of the rectum 24 h before the operation.^30^ This method is simple, inexpensive, and does not require special equipment. In this study, more than 12 LNs were detected in 95.7% of patients who received the nano‐carbon injections. In clinical practice, we found that injecting nano‐carbon suspension under the guidance of an anoscope resulted in clearly stained LNs (Figure 1b), which helped visualize the lateral LNs. Additionally, small LNs were easily detected in the postoperative specimens, improving the detection rate. Although this approach also stained the bowel at the rectal anastomosis (Figure 1c), it did not increase the incidence of anastomotic complications.

For locally advanced rectal cancer (T3−4/N+M0), neoadjuvant therapy based on radiotherapy combined with total mesorectal excision is the standard treatment. Preoperative radiotherapy promotes tumor regression by reducing the positive circumferential and distal surgical margins and the risk of local recurrence and increasing the probability of preserving the anus. The incidence of anastomotic leakage after laparoscopic low rectal cancer surgery is between 10 and 13%. Many factors affect anastomotic healing, such as preoperative radiotherapy and chemotherapy and low anastomosis, which are key risk factors.^31^ A protective stoma after surgery can divert proximal feces, maintaining the anastomosis in a relatively clean state and reducing intestinal pressure, thereby protecting the anastomosis. Regardless of whether it minimizes the incidence of anastomotic leakage, a protective stoma decreases the harm caused by anastomotic leakage.^32^ We observed that nano‐carbon tracer injections under the rectal mucosa caused black staining of the intestinal tube, but it did not affect anastomotic healing. Thus, nano‐carbon tracers help clinicians better visualize the LNs without obvious pain to the patient. During the operation, the LNs stained by the nano‐carbon tracer were clearly visible, significantly increasing the number of detected LNs. Small lymph nodes were also visible, improving the ability of the clinicians to determine the scope of LN dissection during surgery and the ability of the pathologists to identify and detect more LNs. Consequently, clinicians and pathologists can better assess the patient's condition, allowing for more personalized and effective treatment plans, thereby improving the survival rate.

This study had some limitations. Our sample was small and only included patients from a single center. Therefore, more extensive, multi‐center studies should be performed to confirm the efficacy and safety of nano‐carbon tracer injections for LN detection.

Although nanocarbon has been used for the localization of colorectal cancer lesions for many years, there is currently no clear guideline for the use of nanocarbon in colorectal cancer surgery, such as the dosage of injection, the distance between the injection site and the tumor, the injection method, the number of markers, and the preoperative injection time. Recently, Reynolds et al. proposed suggestions for the application of nanocarbon, including injection methods, injection sites, injection indications, and number of labels, but did not mention preoperative injection time, and the effectiveness of implementation still needs further observation. The clinical application of nanocarbon in rectal cancer has not been carried out for a long time and its scope is not broad. There are few evidence‐based literature reports on the application of nanocarbon at home and abroad. Nevertheless, we have reason to expect that with the promotion of the application of nanocarbon, the accumulation of surgeon experience, and the development of standardized surgical methods, nanocarbon will bring benefits to more rectal cancer patients.

In conclusion, submucosal anoscope‐guided injection of nano‐carbon tracers increased the LN detection rate without increasing anastomotic complications in patients with middle and low rectal cancer undergoing laparoscopic anterior resection after neoadjuvant therapy. Therefore, it is a safe and effective method for LN tracing.

## AUTHOR CONTRIBUTIONS

Lelin Pan, Lijun Li designed the study. Feng Shao wrote the original draft. Qi Zhou, Fei Yu collected raw data. Lijun Li performed statistical and bioinformatics analyses. Lelin Pan, Lijun Li supervised the study.

## CONFLICT OF INTEREST STATEMENT

The authors declare no conflicts of interest.

## ETHICAL STATEMENT

This study protocol was approved by the Ethics Committee of the First Affiliated Hospital of Zhejiang University School of Medicine (NO.IIT20220991A).

## INFORMED CONSENT

Informed consent was obtained from all patients.

## Data Availability

The data used to support this study are available from the corresponding author upon request.
